# A Comprehensive Model for Real Gas Transport in Shale Formations with Complex Non-planar Fracture Networks

**DOI:** 10.1038/srep36673

**Published:** 2016-11-07

**Authors:** Ruiyue Yang, Zhongwei Huang, Wei Yu, Gensheng Li, Wenxi Ren, Lihua Zuo, Xiaosi Tan, Kamy Sepehrnoori, Shouceng Tian, Mao Sheng

**Affiliations:** 1 State Key Laboratory of Petroleum Resources and Prospecting, China University of Petroleum, Beijing 102249, P.R. China; 2 Department of Petroleum Engineering, Texas A&M University, Collage Station, TX, 77843, USA; 3 Department of Petroleum and Geosystems Engineering, University of Texas at Austin, Austin, TX, 78712, USA

## Abstract

A complex fracture network is generally generated during the hydraulic fracturing treatment in shale gas reservoirs. Numerous efforts have been made to model the flow behavior of such fracture networks. However, it is still challenging to predict the impacts of various gas transport mechanisms on well performance with arbitrary fracture geometry in a computationally efficient manner. We develop a robust and comprehensive model for real gas transport in shales with complex non-planar fracture network. Contributions of gas transport mechanisms and fracture complexity to well productivity and rate transient behavior are systematically analyzed. The major findings are: simple planar fracture can overestimate gas production than non-planar fracture due to less fracture interference. A “hump” that occurs in the transition period and formation linear flow with a slope less than 1/2 can infer the appearance of natural fractures. The sharpness of the “hump” can indicate the complexity and irregularity of the fracture networks. Gas flow mechanisms can extend the transition flow period. The gas desorption could make the “hump” more profound. The Knudsen diffusion and slippage effect play a dominant role in the later production time. Maximizing the fracture complexity through generating large connected networks is an effective way to increase shale gas production.

Large-scale shale gas production began in 2000, when horizontal drilling and hydraulic fracturing techniques provided access to commercial volumes of shale gas. According to recent EIA report, 43 billion cubic feet of gas per day is produced from shales in the US^1^. Hydraulic fracturing in shale formations is often associated with complex fracture networks^2^,^3^,^4^,^5^,^6^. The occurrence of complex non-planar fracture network is much more common than initially anticipated, especially in unconventional reservoirs^6^. The complexity and non-planarity is caused by the interaction of hydraulic fractures with pre-existing natural fractures, fissures or cleat^4^.

Significant efforts have been made to numerically model shale gas production in complex fracture networks. The dual continuum model (dual porosity and dual permeability)^7^,^8^,^9^,^10^,^11^ and discrete fracture models (DFM)^12^,^13^,^14^ are the two most common methods to handle complex fracture networks and to study flow in fractured reservoirs (with natural fractures and/or induced fractures). Cipolla *et al*. (2011) developed automated unstructured gridding algorithms to numerically simulate well performance from the complex fractures^15^. Li and Lee (2008), Moinfar (2014), used embedded discrete fracture models (EDFM) to treat the matrix as structured grids and discretize the complex fractures into a number of segments^16^,^17^. Sheng *et al*. (2012) integrated a shale-gas transport model with extended finite element method (XFEM) to study the main flow gas mechanism of shale in complex fracture network^18^. Jiang and Younis (2015) proposed two hybrid approaches: one is the coupling EDFM with multiple interacting continua (MINC), the other is the coupling of unstructured DFM with continuum-type approaches^19^. However, these numerical methods are still challenging to apply due to complicated gridding issues, an expensive computational cost, and complexities in development of computational codes.

Analytical and semi-analytical approaches have also been developed to investigate the well performance in complex fracture networks. Zhou *et al*. (2013) proposed a semi-analytical model by combining an analytical reservoir solution with a numerical solution on a discretized fracture panels^20^. However, the model did not incorporate the main gas transport mechanisms in shale gas formations. Yu *et al*. (2015) developed a comprehensive semi-analytical model for gas transport in shale formation with complex fracture geometry^21^. The model considered the complex non-planar fractures with varying fracture width and fracture permeability, and the gas transport mechanisms in shale. However, there is no systematic studies for the effects of various gas flow mechanisms on the production. Besides, the complex fracture network due to the interconnection of hydraulic fracture and natural fractures has not been considered. Jia *et al*. (2015) adopted the Star-Delta transformation to solve the interplay of flow between the interconnected fractures in their semi-analytical model^22^. However, the method is based on the discrete fracture network simulation and the fracture flow is numerically solved by the method of finite difference method, which is also related to gridding problem and computational cost. Moreover, most of the recent models only solved the orthogonal fracture network without considering the arbitrary fracture orientation and geometry^22^,^23^.

Furthermore, the common methods for analysis of different gas flow regimes is mainly focused on the transient pressure behavior under constant flow rate^24^,^25^,^26^, because most of these models are within the Laplace domain, which are proved to be acceptable for fractures with infinite conductivity. However, shale-gas wells with low permeability and finite flow capacity fractures are generally produced at constant bottomhole pressure rather than constant flow rate^27^,^28^. Accordingly, type curves under constant bottomhole pressure, i.e. rate transient analysis, is especially more useful to identify the flow regime and deserves more interest to estimate the fracture properties.

In this paper, we develop a comprehensive and efficient semi-analytical model by incorporating the main shale gas flow mechanisms, including gas diffusion, gas desorption, gas slippage, and non-Darcy’s flow in the complex fracture network. An innovative approach defined as “Correction of Flow Performance at Interconnected Nodes” is introduced to consider the interplay of flow between the interconnected fractures. Then, we verify the semi-analytical model against a numerical model and an analytical model. Subsequently, the effects of various gas flow mechanisms and fracture network complexities on well performance and rate transient behavior with the constraint of constant bottomhole pressure are studied systematically. Furthermore, we apply the model to perform history matching and production forecasting in an actual vertical fractured well from Marcellus shale. The semi-analytical model we present is simple-yet-rigorous to deal with complex fracture network with arbitrary orientation, geometry, various properties and interconnections between fractures. Besides, by use of the varying time step automatically according to the changing speed of gas flow rate, the model is computationally efficient. To best of our knowledge, this work is the first study that presents the impacts of gas transport mechanisms on well performance and rate transient analysis in shale formations with the complex non-planar fracture network.

## Results

### Model validation

**Figure 1:**
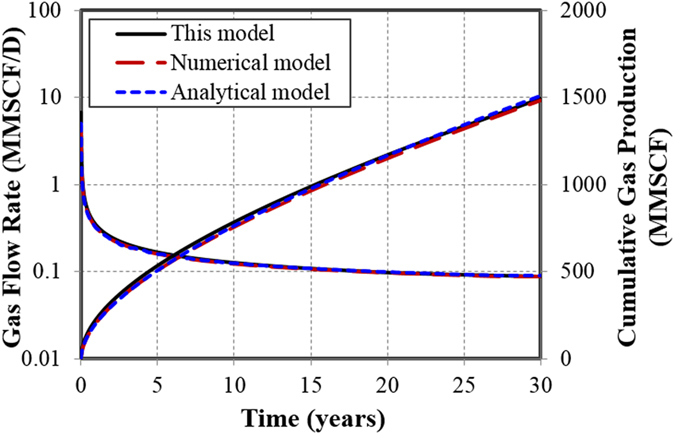
Model validation with numerical model and analytical model.

The accuracy of the semi-analytical model is confirmed by comparing the results with the numerical simulation (CMG, 2015) and an analytical solution (Kappa, 2015). The reservoir and fracture properties are as follows: initial reservoir pressure is 5,000 psi, reservoir temperature is 130 °F, reservoir permeability is 500 nd, reservoir porosity is 7%, reservoir thickness is 150 ft, rock compressibility is 1 × 10^−6^ psi^−1^, fracture width is 0.01 ft, fracture half-length is 350 ft, fracture conductivity is 50 md-ft, Langmuir pressure is 1,300 psi, Langmuir volume is 140 scf/ton, and shale bulk density is 2.5 g/cm^3^. The constant bottomhole pressure of 500 psi is used for simulation constraint and the simulation time is 30 years. Figure 1 shows a good match of gas flow rate and cumulative gas production between the semi-analytical model and numerical model and analytical model.

### Effects of gas flow mechanisms

Shale gas is natural gas produced from shale sequences^29^. Due to low permeability of the shale rock (nano-Darcy scales) and organic matter as a medium of gas source and storage, the gas transport mechanism is significantly different from conventional natural gas^30^. We will investigate the physical aspects of gas production from shale with a single bi-wing fracture in the following sections.

### Gas diffusion

According to Javadpour *et al*. (2007), the gas evolution process from shale has four different transport processes: (1) gas flow in micro-pores, i.e. gas flow in fractures, which can be described by Fickian diffusion and/or Darcy’s law depending on the original pressure; (2) gas flow in nano-pores, i.e. gas flow in shale matrix, where a Knudsen diffusion is the dominant diffusion process; (3) Gas desorption from the surface of the kerogen/clays to the pore networks; (4) Gas molecule diffusion from the kerogen bulk or clays to the exposed surfaces^29^. Besides, the experimental studies show that the diffusion coefficient for Knudsen diffusion is 4 × 10^−2^ cm^2^/s and the diffusion coefficient for gas molecule transport in the kerogen bulk is 2 × 10^−6^ cm^2^/s^29^. Kim *et al*. (2015) presented that the Fick diffusion coefficient remains 5.068 × 10^−4^ cm^2^/s and is independent of the pore radius^31^. Therefore, in this study, the range of diffusion coefficient is determined from 1 × 10^−2^ to 1 × 10^−4^ cm^2^/s.

**Figure 2:**
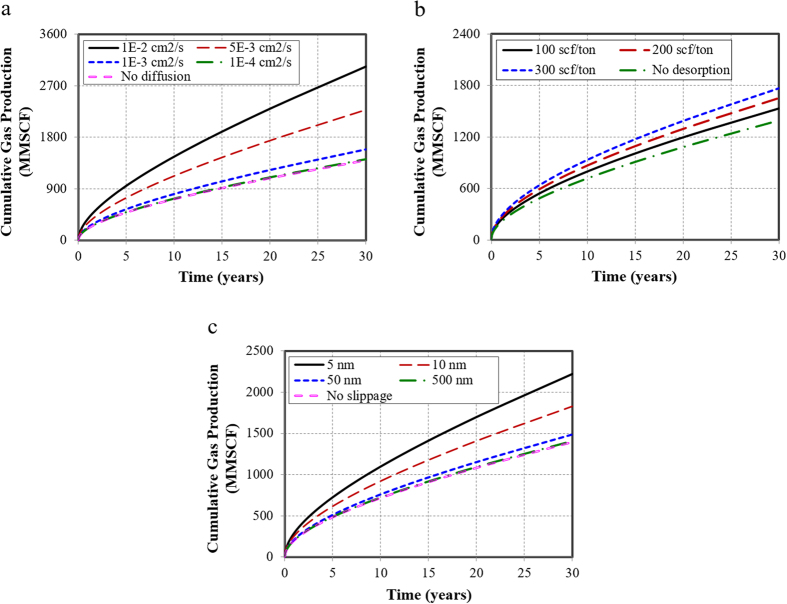
Effects of three gas transport mechanisms on cumulative gas production. (**a**) Effect of gas diffusion with different gas diffusion coefficients. (**b**) Effect of gas desorption with different Langmuir volumes. (**c**) Effect of gas slippage with different pore diameters.

The simulation results show that the higher the diffusion coefficient, the greater the diffusion effect is, especially when it is larger than 1 × 10^−3^ cm^2^/s, the impact increases drastically (Fig. 2a). For the diffusion coefficient of 1 × 10^−2^ cm^2^/s, which is within the scope of Knudsen diffusion, the contribution to cumulative production is up to 54%. Hence, Knudsen diffusion plays a dominant role in the gas production. When the diffusion coefficient is lower than 1 × 10^−4^ cm^2^/s, the gas diffusion plays a negligible role in well performance and the contribution to well performance is only 0.01%.

### Gas desorption

Gas adsorption and desorption is an important process in organic rich shale reservoirs^32^. The organic matter has a strong adsorption ability because of the large surface area and affinity to methane^21^. Gas is supposed to first desorb from the surface of nano-pores to matrix then transport into fractures. Different gases have different Langmuir adsorption capacities^32^.

The effects of different Langmuir adsorption capabilities on well performance are shown in Fig. 2b. It suggests that the gas desorption contributes to 10–27% increase of cumulative gas production at 30 years. This is because gas desorption increases the effective pore diameter for flow, reduces tortuosity and causes extra slippage at the boundary, thereby increases the matrix permeability manifold^33^. In addition, the production is higher with a larger value of Langmuir volume. The reason is that the Langmuir volume reflects the capacity of adsorbed gas in the reservoir. The larger the value, the more adsorbed gas in the reservoir, and the more gas could desorb from matrix to fractures when well produces at a constant bottomhole pressure.

### Gas slippage

Gas flows through shale matrix with pore size ranging from nanometers (1 nm = 10^−9^ m) to micrometers (1 μm = 10^−6^ m)^29^. The velocity of gas molecules at pore walls is referred to the gas slip velocity. Because of the comparable dimensions of pore size in a shale reservoir to the mean free path of molecules, the slip velocity is not zero^34^. This phenomenon is known as gas slippage effect or Klinkenberg effect^35^. Small pore size enhances gas slippage along pore walls and needs to be quantified^36^. To illustrate the gas slippage effect on cumulative gas production and rate transient behavior, the range of pore size from 5 nm to 500 nm is studied.

Simulation results indicate that the smaller the pore size is, the more important the gas slippage effect (Fig. 2c). When the pore size is 5 nm, the slippage effect could contribute to 37% of increase in cumulative gas production. When the pore size approaches 500 nm, the gas slippage effect becomes negligible, which contributes only 0.69% of the increase in cumulative gas production.

The reason is that the smaller pore size results in a higher Knudsen number under the same pressure and permeability condition. A higher Knudsen number indicates the distances between gas molecules are comparable to the pore dimension, resulting in rarefied gas^34^,^37^, thus more gas production could be obtained. Besides, the slippage effect could accelerate the gas molecules transport speed because there is less drag or no stationary layer to slow them^34^. Therefore, slippage effect, acting as an enhancement of apparent permeability, could increase the shale gas production.

### Effects of fracture complexity

**Figure 3:**
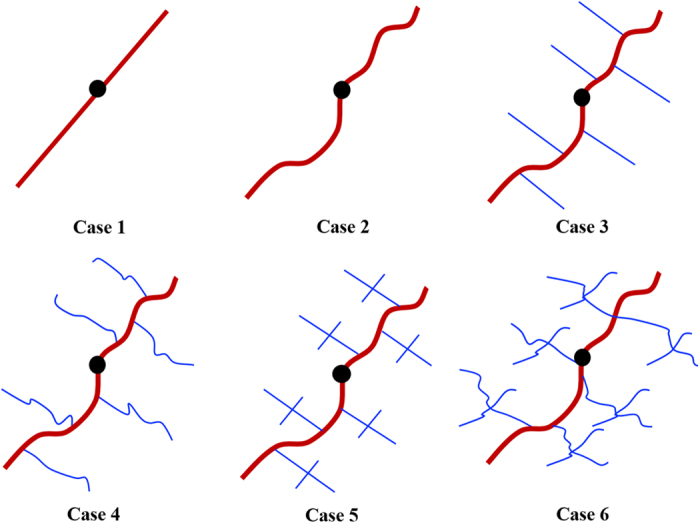
Six different complex fracture networks. Case 1 is the simple planar hydraulic fracture; Case 2 is the non-planar hydraulic fracture; Case 3 is the simple planar natural fracture; Case 4 is the non-planar natural fracture; Case 5 is the simple planar natural fracture network; Case 6 is the non-planar natural fracture network.

The effects of complex fracture networks on well performance are studied. The fractures are simulated from simple to complex. Six cases are investigated to illustrate the fracture complexity (Fig. 3). Case 1 is simple planar hydraulic fracture without considering natural fractures. Case 2 is non-planar hydraulic fracture without considering natural fractures. Case 3 is non-planar hydraulic fracture interconnected with simple planar natural fractures. Case 4 is non-planar hydraulic fracture interconnected with non-planar natural fractures. Case 5 is based on Case 3, but with more complicated natural fracture networks, i.e. the planar natural fractures are also interconnected with each other. Case 6 is based on Case 5, the planar natural fractures are treated as non-planar natural fractures. The natural fracture properties are as follows: fracture half-length is 180 ft for the ones interconnected with hydraulic fracture and 100 ft for natural fractures in the network. Fracture width and fracture conductivity is 0.01 ft and 1 md-ft for both of the natural fracture systems.

### Non-planar hydraulic fracture

To illustrate the effect of non-planar hydraulic fracture on gas production, Cases 1 and 2 are compared. In this situation, only one single bi-wing hydraulic fracture is considered. The total fracture length is the same for both cases. The difference is that Case 1 is a straight hydraulic fracture, while Case 2 has an arbitrary geometry.

**Figure 4:**
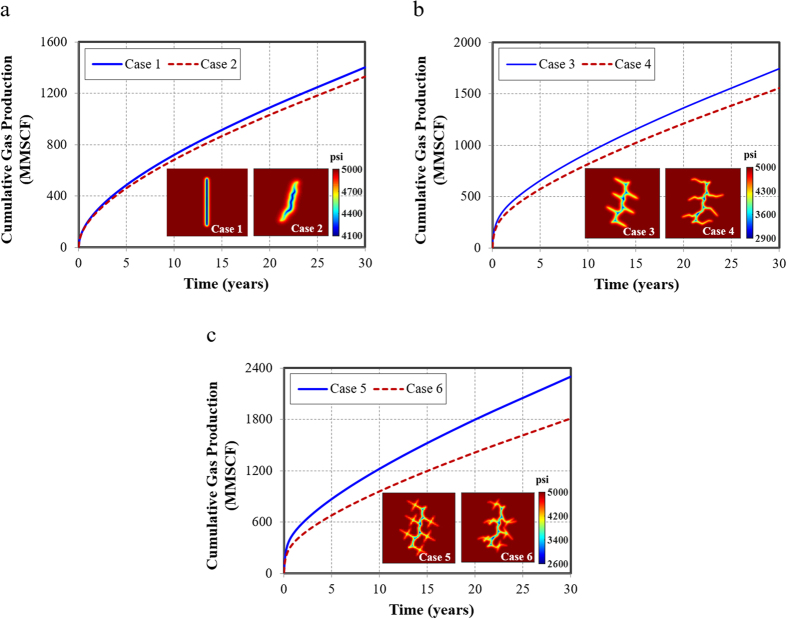
Effects of fracture complexity on gas production and pressure distribution after 10 days. (**a**) Effect of non-planar hydraulic fracture; (**b**) Effect of non-planar natural fracture; (**c**) Effect of non-planar natural fracture network.

As illustrated by Fig. 4a, there is 5% higher of cumulative gas production for the simple planar hydraulic fracture. Hence, the planar hydraulic fracture could overestimate the cumulative gas production. The possible reason could be that fracture segments in non-planar fracture have a larger production interference and competition with each other, which could be regarded as a reduction of fracture conductivity. The pressure distribution after 10 days production suggests that the drainage area of the non-planar fracture is smaller than the simple planar fracture, resulting in the smaller pressure drop, thus less gas production was obtained.

### Non-planar natural fracture

Shale gas reservoir is the naturally fractured formation. The interaction between hydraulic fracture and natural fractures is common. Cases 3 and 4 are compared to study the effect of non-planar natural fractures.

As indicated in Fig. 4b, hydraulic fracture interconnected with simple planar natural fractures could overestimate about 12% of cumulative gas production. Similar with the effect of non-planar hydraulic fracture, with the appearance of interconnection between hydraulic fracture and natural fractures, the significance of non-planar natural fracture becomes more pronounced. The reason could be attributed to the occurrence of more production interference among the fractures including hydraulic fracture with natural fractures and natural fractures with each other.

### Non-planar fracture network

In this simulation case, the natural fractures are also interconnected with each other, the effect of non-planar natural fracture networks is studied.

As shown in Fig. 4c, hydraulic fracture is interconnected with simple planar natural fracture networks could overestimate about 21% of cumulative gas production. Similarly, the significance of non-planar natural fracture network is increasingly noteworthy. Therefore, with the increasing complexity of the fracture network, the impact of non-planar fracture increases, indicating that it is important to accurately characterize the realistic complexity of the fracture network in actual field application.

The results show that the more complex the fracture network is, the higher the production could be obtained. The hydraulic fracture interconnected with natural fracture networks could achieve the highest gas recovery at the end of production. Comparing Case 2 with Case 4, the natural fracture contributes to 17% of cumulative gas production. Comparing Case 4 with Case 6, the natural fracture network could contribute to 36% of cumulative gas production. Therefore, increasing the fracture complexity and the interconnections between hydraulic fracture and natural fractures could improve the gas production significantly.

Maximizing the fracture complexity through generating large fracture networks is an effective way to increase shale gas production. This can be done through pumping large volumes of low viscosity fluid, for example, slick water. In addition, low viscous fluid could improve the clean-up behavior^4^.

### Field application

One vertical well in Marcellus shale is selected to perform history matching and further illustrate the application of this semi-analytical model. The reservoir and fracture properties are as follows: the initial reservoir pressure is 4,917 psi, the reservoir temperature is 130 °F, the reservoir porosity is 6.8%, the reservoir thickness is 80 ft, the rock compressibility is 3 × 10^−6^ psi^−1^, the initial gas saturation is 75%, and the gas gravity is 0.59.

Based on the given parameters, the semi-analytical model is used to perform history matching and production forecasting. The bottomhole pressure of 500 psi is used for simulation constraint and cumulative gas production is the history-matching variable. Complexity of fracture networks, fracture half-length, fracture conductivity, and matrix permeability are tuning parameters to perform history matching. The Langmuir volume is 140 scf/ton, the average pore size is 10 nm, and the diffusivity coefficient is 4 × 10^−2^ cm^2^/s.

Because the history-matching process usually leads to non-unique solutions. In other words, different sets of values can achieve satisfactory match results. Due to lack of data for this well such as microseismic monitoring, advanced sonic logs, 3D-seismic interpretation of curvature stress and natural-fracture orientation, two possible history-matching results are generated below: one is the planar fracture network and the other one is the non-planar fracture network. The geometry of non-planar fracture network is mainly referred to the mechanical analysis of interaction between hydraulic and natural fractures in shale gas reservoir^38^. Both of the fracture network have the same fracture properties and total fracture length. The uncertainty quantification and integration of history matching with microseismic data will be further investigated in our future work.

**Figure 5:**
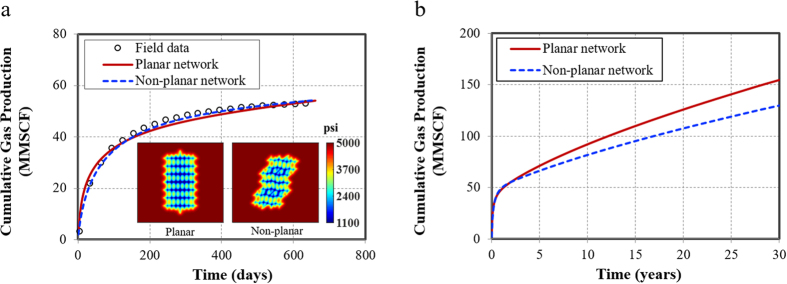
History matching and production forecasting for an actual vertical well from Marcellus shale. (**a**) History matching results for both planar fracture network and non-planar fracture network and pressure distribution after 30 years production. (**b**) Comparison of cumulative gas production for a 30-year period.

The history matching results for cumulative gas production are shown in Fig. 5a, which illustrates a good match between both of the planar fracture network and non-planar fracture network with the field data. In addition, the pressure distribution for both cases at production time of 30 days clearly indicates that the effective gas drainage area is different between two cases. For the planar fracture network, the primary drainage area is the vicinity of the fractures near the wellbore. While for the non-planar fracture network, the interaction area between the hydraulic fracture and natural fractures is the primary drainage area, illustrating the importance of interference of fractures inside the network.

Through the history matching, the matrix permeability, fracture network complexity and fracture properties can be possibly quantified as follows: the matrix permeability is 50 nd; the fracture network consists of one bi-wing hydraulic fracture and 98 natural fractures, among them, 14 natural fractures are interconnected with the hydraulic fractures. The fracture width is 0.01 ft for all the fractures in the network. The fracture half-length is 200 ft, 100 ft and 25 ft for hydraulic fracture, natural fractures interconnected with hydraulic fractures, and natural fractures in the network, respectively. The fracture conductivity is 5 md-ft and 1 md-ft for hydraulic fracture and natural fractures, respectively.

After history matching, we performed production forecasting for a 30-year period using both planar fracture network and non-planar fracture network. Although at early production time, these two networks obtained the same amount of cumulative gas production. However, after about 2.25 years, the fracture interference starts to play increasing important roles in the gas production performance, resulting in difference between two networks. At the end of 30 years, there is 19% difference for the cumulative gas production (Fig. 5b). The planar fracture network could overestimate well performance because of less fracture interference. Hence, it is extremely important to characterize the realistic fracture networks underground for accurate long-term production prediction. The findings of the field application may emphasize the importance of characterizing the realistic complexity of the fracture network and their properties.

## Discussion

The gas flow mechanisms and fracture complexity have significant impacts on the cumulative gas production. Rate transient analysis and flow regime identification are investigated to further explain the reasons. The reservoir, fracture and gas properties are adopted from the field case study discussed above.

### Effect of fracture network on rate transient behavior

In this study, the single bi-wing hydraulic fracture without considering natural fractures is the reference case. The total fracture length remains the same as the fracture network.

**Figure 6:**
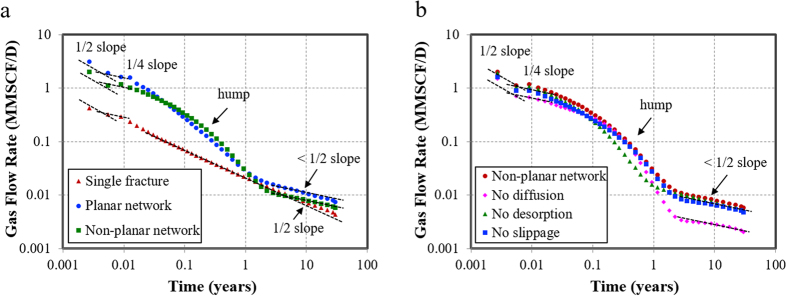
Effects of fracture complexity and gas flow mechanisms on rate transient behavior. (**a**) Effects of fracture complexity and non-planar network on rate transient behavior. (**b**) Effects of gas diffusion, gas desorption and gas slippage on rate transient behavior.

The gas flow rate versus production time on a log-log scale is plotted to identify the flow regimes experienced by the well (Fig. 6a). For the early production time, most of the gas entering the wellbore comes from the expansion of the system within the fracture and flow is essentially linear. The log-log graph of flow rate against time yields a straight line with a slope of 1/2. After the fracture linear flow regime, two linear flows occur simultaneously. One flow is linear flow within the fracture and the other is in the formation, thus the bi-linear flow is with a 1/4-slope straight line. When the fracture tip effects are felt at the wellbore, bi-linear flow periods end and formation linear flow begins. The gas flow becomes formation linear flow with a 1/2-slope straight line.

However, with the appearance of natural fractures, a “hump” could be characterized at the transition flow regime between bi-linear flow and formation linear flow. Besides, at later production time, i.e. the formation flow period, the slope is less than 1/2, suggesting a relatively higher gas rate with the contribution from natural fractures. This phenomenon could possibly help field analysis to identify the complexity of the fracture network and reveal whether there is a good connection between hydraulic fractures and natural fractures. The “hump” indicates the interference between fractures. The sharper of the “hump”, the more severe of the interference, thus more complex the fracture network will be. Furthermore, the presence of non-planar fracture network also plays an important role in the transition period. Gas flow rate is higher for the non-planar fracture network than the planar fracture network during this period. In addition, a sharper “hump” could be obtained than the planar fracture network, suggesting stronger fracture interference for the non-planar fracture network. Combined with gas flow rate or cumulative gas production and the sharpness of the hump could possibly assist in the field analysis to identify whether there is a severe irregularity of the fracture network.

### Effects of gas flow mechanisms on rate transient behavior

In this study, the non-planar fracture network is the reference case, because we want to verify the effects of gas flow mechanisms under the complex non-planar fracture network. Cases without considering gas diffusion, gas desorption and gas slippage are compared and analyzed.

Figure 6b presents that all the gas flow mechanisms could extend the transition flow period, especially the gas desorption. This is because at early production time, free gas releases fast from matrix to the fractures. With the depletion of reservoir pressure, gas desorbs from the matrix particles to matrix pores to compensate the reduction of the pressure. Therefore, at the transition flow period, gas desorption plays a major role in the gas production. Furthermore, gas desorption could make the “hump” more profound. Gas slippage and gas diffusion mainly influence the gas rate at later production time, especially the Knudsen diffusion dominates the flow behavior in the formation linear period. The possible reason could be that with the continuous decreasing reservoir pressure, the Knudsen number increases, the slippage effect and Knudsen diffusion tend to play a major role in the nano-scale flow perspective, and the viscous pipe flow is almost negligible^39^,^40^.

## Method

### Model Development

The semi-analytical model combines an analytical reservoir solution with a numerical solution on the discretized fracture segments. The major assumptions are:

- The reservoir is homogeneous and isotropic with uniform thickness and constant porosity permeability and compressibility;
- The fluid flow is single gas phase;
- The height of each fracture is equal to that of reservoir;
- The gravity force is neglected.

### Shale gas flow in matrix system

The diffusivity equation for shale gas transport in the matrix can be expressed below:


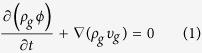


where *ρ*_*g*_ is gas density, *ϕ* is matrix porosity, *v*_*g*_ is gas velocity. Taking the gas slippage and gas diffusion into account, the gas velocity can be expressed as^21^:


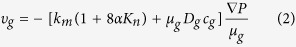


where *k*_*m*_ is matrix permeability, *α* is a constant and close to 1, *K*_*n*_ is Knudsen number, *μ*_*g*_ is gas viscosity, *D*_*g*_ is diffusion coefficient, *c*_*g*_ is compressibility of real gas.

The gas desorption effect is considered by revising the gas compressibility as following:


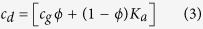


where *K*_*a*_ is the differential equilibrium portioning coefficient of gas at a constant temperature, which could be expressed as following^21^:


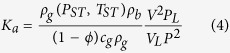


where *V*_*L*_ is Langmuir volume, and *P*_*L*_ is Langmuir pressure. *P*_*ST*_ and *T*_*ST*_ are pressure and temperature at standard condition.

Therefore, the diffusivity equation of gas transport in shale matrix is given by:


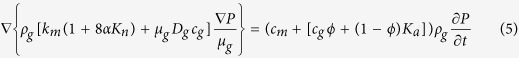


where *c*_*m*_ is matrix rock compressibility.

For real gas flow, pseudo pressure is introduced^41^:


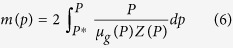


where P^*^ is the reference pressure, *Z* is the gas compressibility factor, dimensionless, which can be obtained through the relationship with pseudo reduced pressure and pseudo reduced temperature^42^,^43^:


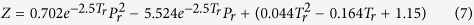



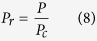



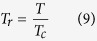


where *P*_*r*_ is pseudo reduced pressure, dimensionless; *T*_*r*_ is pseudo-reduced temperature, dimensionless; *P*_*c*_ is critical pressure of gas; *T*_*c*_ is critical temperature of gas.

The gas density can be estimated by the following formula:


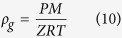


where *M* represents the gas molecular weight (*M* = *M*_*air*_*γ*_*g*_, where *M*_*air*_ is the air molecular weight and equals to 29 g/mol),*R* = 8.1345*kPa* × *m*^3^(*kmoles/K*) is the general gas constant, and *T* is the absolute temperature.

The gas viscosity is given by^44^:


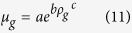



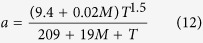



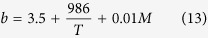



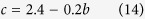


Then, the gas compressibility can be determined as follows:


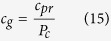


where *c*_*pr*_ is reduced gas compressibility, which can be estimated by:


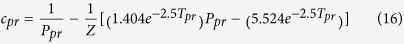


### Shale gas flow in the fracture network

Gas flow from fracture to the wellbore is described by Darcy’s law. Due to the high gas velocity, especially at early production time, non-Darcy flow is used to model the additional pressure drop. Pressure drop is given as followings when gas flow from *j*+*1*th node to *j*th node by Zhou *et al*. (2014)^20^:


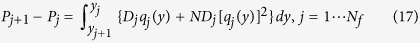



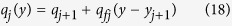



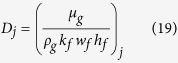



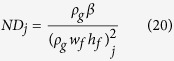


where *P*_*j*_ is the gas pressure at *j*th node, *q*_*fj*_ is the gas flux at *j*th fracture segment, *q*_*j*_ is the gas flow rate at *j*th node of fracture segment, *D*_*j*_ is the coefficient of Darcy flow for gas at *j*th fracture segment, *k*_*f*_ is the fracture permeability, *w*_*f*_ is the fracture width, *h*_*f*_ is the fracture height, *ND*_*j*_ is the coefficient of non-Darcy flow, and *β* is the non-Darcy Forchheimer coefficient.

## Model solution

**Figure 7:**
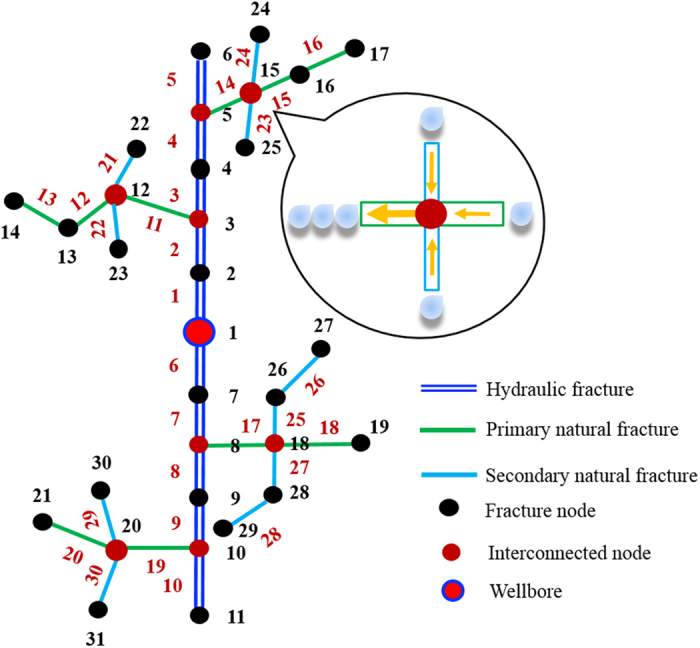
An example of fracture network discretization. The fracture network consists of one hydraulic fracture, four primary natural fractures and ten secondary natural fractures. The hydraulic fractures are connected with natural fractures, and natural fractures are also connected with each other. The wellbore is in the middle of the figure. The fracture network is discretized into 31 nodes (indexed in black) and 30 segments (indexed in red).

**Figure 8:**
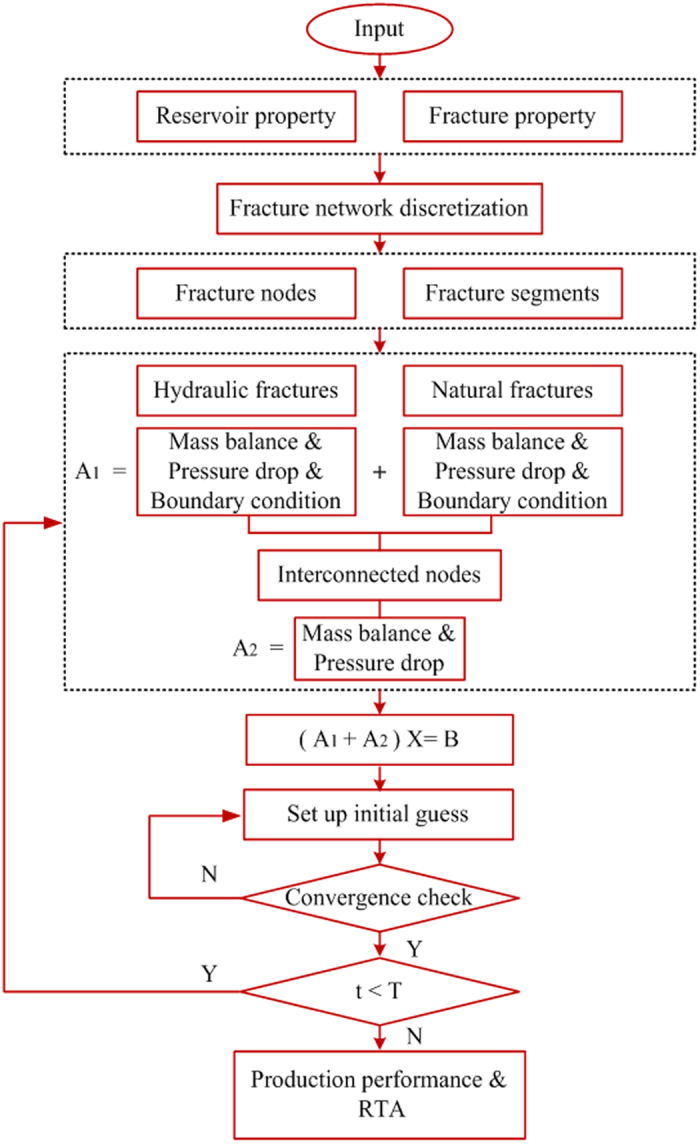
Flow chart of the calculation procedure.

The fracture network can be divided into *N* groups system depending on the complexity. Figure 7 shows an example of the fracture network. The hydraulic fracture is in the middle of blue color. Others are natural fractures. The natural fractures interconnected with hydraulic fracture are called “Primary natural fracture system”, the fractures interconnected with “Primary natural fracture system” are called “Secondary natural fracture system.” If there are other natural fractures interconnected with “Secondary natural fracture system”, they can be named as “Tertiary natural fracture system.” The calculation method can be followed five steps and a flow chart is shown in Fig. 8.

- Step 1: Fracture discretization and numbering
- The fracture network is discretized into a number of segments and the associated nodes connecting these segments, which should capture the topology and fracture characteristics. The fracture segments and nodes could be numbered following the sequence of hydraulic fracture, primary, secondary, tertiary natural fractures. Figure 7 is an example of the discretization of the fracture network. The fracture network consists of single bi-wing hydraulic fracture, 4 primary natural fractures and 10 secondary natural fractures. The network is discretized into 30 fracture segments with 31 nodes.
- Step 2: Equation system set up at each fracture node:

Two equation systems are introduced at the fracture nodes:

1. Mass balance. Inflow should equal to outflow at each node.
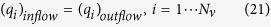
where (*q*_*i*_)_*inflow*_ is the gas inflow rate at *i*th node, (*q*_*i*_)_*outflow*_ is the gas outflow rate at *i*th node.
2. Pressure drop.

The pressure drop at each node is calculated by equations (17) considering non-Darcy flow. Thus the equation system is non-linear.

- Step 3: Boundary conditions implementation

The boundary condition of the equation system is the coupling of the flow from shale matrix with fracture flow. Pressure at any point of each fracture segment obtained from equation (5) is the same as the result obtained from equation (17).


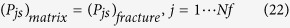


where *P*_*js*_ represents pressure at any point of each fracture segment.

For gas flow in the fracture, Eq. (17) is used. For gas transport in shale matrix, the unsteady state gas diffusivity equation [Eq. (5)] is solved analytically based on the point source solutions in real domain presented by Gringarten and Ramey (1973)^45^. Each fracture segment can be treated as a plane source. The pressure response at any point in the reservoir can be obtained through the superposition principle of all fracture segments. The plane source pressure drop in 2D infinite space of reservoir point (x, y) can be expressed below^46^:


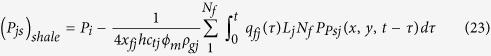



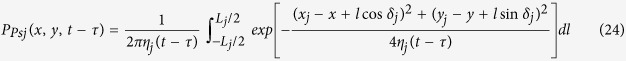


where *η*_*j*_ is reservoir diffusivity for *j*th segment, and is suggested to be revised by shale gas flow mechanism, 
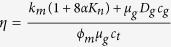
, *c*_*t*_ is the total compressibility (*c*_*t*_=*c*_*m*_+*c*_*d*_), *δ*_*j*_ is the angle between the *j*th fracture segment and the x-axis.

The “planarity” of the fracture network is mainly controlled by the number of discretized fracture segments in step 1 and angle input in step 3. One can discretize the fracture network into any numbers of segments at any angle according to the complexity of the fracture network. Therefore, an arbitrary fracture geometry can be simulated and the planarity of the fracture network can be controlled in such a simple way by using this semi-analytical model.

- Step 4: Solve non-linear system of equations using the Newton-Raphson algorithm.

The interconnection of the fractures is calculated by an innovative approach defined as “Correction of Flow Performance at Interconnected Nodes”:


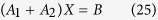


*A*_*1*_ is the system of equations within each network of fractures. The mass balance equation, pressure drop equation and boundary conditions are adopted for each node and segment within their own fracture system without considering the interconnections between other fractures.

*A*_*2*_ is the correction of flow rate and pressure drop at the interconnected nodes. The interconnected nodes are input, their flow rate and pressure drop are corrected by the interconnected fracture segments: the flow rate contribution from the interconnected fracture segment is added to the interconnected nodes, then the pressure drop equations are added correspondingly. The calculation flow chart is illustrated the process in detail.

- Step 5: Automatic variation time step.

Variation of time step very often used to maximize the computational speed. We select the time step according to the changing extent of gas flow rate. If the change of gas flow rate is minor, i.e. within a predetermined tolerance scope, the time step will become larger automatically. Therefore, in the early production time, the gas flow rate changes dramatically, the time step is smaller and more iteration steps need to be taken. While, the situation is just the opposite at later production time, thus less iteration steps are needed.

## Additional Information

**How to cite this article**: Yang, R. *et al*. A Comprehensive Model for Real Gas Transport in Shale Formations with Complex Non-planar Fracture Networks. *Sci. Rep.* **6**, 36673; doi: 10.1038/srep36673 (2016).

**Publisher’s note**: Springer Nature remains neutral with regard to jurisdictional claims in published maps and institutional affiliations.
